# The role of angiotensin-converting enzyme 2 (*ACE2*) genetic variations in COVID-19 infection: a literature review

**DOI:** 10.1186/s43042-022-00309-6

**Published:** 2022-05-28

**Authors:** Manal S. Fawzy, Hend Ashour, Aya Allah Ashraf Shafie, Nesrine Ben Hadj Dahman, Abdelhamid M. Fares, Sarah Antar, Ahmed S. Elnoby, Fatma Mohamed Fouad

**Affiliations:** 1 Department of Medical Biochemistry and Molecular Biology, Faculty of Medicine, Suez Canal University, Ismailia, Egypt; 2 Department of Medical Physiology, Faculty of Medicine, King Khalid University, Abha, Saudi Arabia; 3 Department of Medical Physiology, Faculty of Medicine, Cairo University, Giza, Egypt; 4 Chemistry/Biochemistry, Faculty of Science, Cairo University, Giza, Egypt; 5 Faculty of Medicine of Tunis, University of Tunis El Manar, 2000 Bardo, Tunis, Tunisia; 6 Department of Pathology, Faculty of Veterinary Medicine, University of Sadat City, Fifth Zone, Ministries Complex, Sadat City, 32511 Menoufia Egypt; 7 College of Veterinary Medicine, Yangzhou University, Yangzhou, 225009 China; 8 Medical Biochemistry Department, Faculty of Medicine, Mansoura University, Mansoura, Egypt; 9 Clinical Pharmacy Department, Children’s Cancer Hospital Egypt, Cairo, 57357 Egypt; 10 Biotechnology/BioMolecular Chemistry, Faculty of Science, Cairo University, Giza, Egypt; 11 Safaga, Red Sea, Egypt

**Keywords:** ACE2, COVID-19, Genetic variants, SARS-CoV2

## Abstract

**Background:**

The angiotensin-converting enzyme-2 (*ACE2*) is recognized to be the fundamental receptor of severe acute respiratory syndrome coronavirus-2 (SARS-CoV2), responsible for the worldwide Coronavirus Disease-2019 (COVID-19) epidemic. However, genetic differences between people besides racial considerations and their relation to disease susceptibility are still not fully elucidated.

**Main body:**

To uncover the role of *ACE2* in COVID-19 infection, we reviewed the published studies that explore the association of COVID-19 with the functional characteristics of *ACE2* and its genetic variations. Notably, emerging studies tried to determine whether the *ACE2* variants and/or expression could be associated with SARS-CoV/SARS-CoV2 have conflicting results. Some researchers investigated the potential of “population-specific” *ACE2* genetic variations to impact the SARS-CoV2 vulnerability and suggested no ethnicity enrichment for *ACE2* polymorphisms that could influence SARS-CoV2 S-protein binding. At the same time, some studies use data mining to predict several *ACE2* variants that could enhance or decline susceptibility to SARS-CoV. On the other hand, fewer studies revealed an association of *ACE2* expression with COVID-19 outcome reporting higher expression levels of *ACE2* in East Asians.

**Conclusions:**

*ACE2* gene variants and expression may modify the deleterious consequences of SARS-CoV2 to the host cells. It is worth noting that apart from the differences in gene expression and the genetic variations of *ACE2*, many other environmental and/or genetic factors could modify the disease outcome, including the genes for the innate and the adaptive immune response.

## Background

More than 24 months have passed since the first discovery of the novel severe acute respiratory syndrome coronavirus-2 (SARS-CoV2) cases in Wuhan, Hubei Province, China. However, it is still spreading enormously, causing a significant health issue in nearly all countries around the world, even those who have already confined the disease spread still worry about having many other waves. From the experience of the previous epidemics, understanding details of the disease pathophysiology could help by a significant way in its handling and control strategies, which we are in dire need to stop world losses from this pandemic. Although previous reports characterized the elderly age group as a risk factor for COVID-19, in particular, if associated with chronic diseases such as hypertension, heart disease, and/or diabetes mellitus [1], nowadays increasing the number of young cases with early complications necessitating ICU and multivisceral support becomes devastating which support the potential contribution of the genetic factors to this risk that warranted continuous research [2].

Given the essential roles of the renin-angiotensin system (RAS) in maintaining the balance of lung cell proliferation/apoptosis and mediating the intra-pulmonary blood pressure, inflammation, and fibrosis, its dysregulation has been linked to several pulmonary diseases, including COVID-19 [3, 4]. The angiotensin-converting enzyme 2 (ACE2), the homolog of ACE, is a catalytic component of RAS that has recently attracted global recognition [5]. ACE2 is reported to be the fundamental entry point of SARS-CoV [6]. It is required for host cell entry and subsequent viral replication after priming by the serine protease TMPRSS2 (transmembrane protease, serine 2) [7], as detailed in the following sections. Despite the spike proteins of SARS-CoV-2 and SARS-CoV are not identical, SARS-CoV2 spike protein has a much higher binding affinity to human ACE2 [8] and supports intense interaction with it [9], which signifies its enhanced pathogenicity.

Rather than the pulmonary expression, ACE2 is reported to be also distributed in the heart, the renal and luminal surface of intestinal epithelial cells, among others [10], explaining the SARS-CoV2 entry site in Wuhan patients and the multi-organ dysfunction observed in infected patients [11]. By using normal lung tissues, Zhao et al. have detected that about 83% of the lung ACE2 expression is situated in the alveolar epithelial cells type II, which may facilitate coronaviral invasion and harbor the virus for replication [12].

Accumulating evidence indicates that *ACE2* genetic polymorphisms among populations and racial considerations may correlate with cellular susceptibility to SARS-CoV2 infection with controversial findings [13–15]. Also, the rationale for the genetic basis of ACE2 or coronavirus-resistant *ACE2* mutant receptors is still mostly unknown in different populations. In this sense, this review aimed to explore some basics related to ACE2-SARS-CoV2 interaction and cell entry and the relation of different *ACE2* variants to disease risk, severity, and progression among different populations worldwide.

## Main text

### Methods

We screened the following medical electronic databases: PubMed, Web of Science, Scopus, and Cochrane CENTRAL for the relevant published data up to February 2022, using the keywords (“COVID-19” OR “SARS-CoV-2” OR “Coronavirus” OR “severe acute respiratory syndrome coronavirus-2” OR “coronavirus SARS-CoV-2” OR “2019-nCoV”) AND (“ACE II receptors” OR “angiotensin-converting enzyme 2” OR “angiotensin-converting enzyme-2” OR “ACE2” OR “angiotensin II receptor blockers” OR “Angiotensin-converting enzyme inhibitors” OR “ACE inhibitors”) AND (“Genetic Variations” OR “Genetic Diversity” OR “SNP” OR “polymorphism” OR “genotype” OR “single nucleotide polymorphism”). The identified records evaluated against the following inclusion criteria: studies are exploring the association of COVID-19 with *ACE2* genetic variations, all types of studies, and the studies published in both peer-reviewed journals and as a preprint.

Different databases were applied to explore the structural and functional characteristics of the *ACE2* gene. The data for gene structure and transcript splicing variant were obtained from Ensembl (www.ensembl.org). Predicted sequence of ACE2 protein and the essential structural motifs and the amino acid residues (in particular the amino acids required for virus binding) with their mutation outcomes were obtained by UniProt (https://www.uniprot.org/uniprot/Q9BYF1/). Protter (http://wlab.ethz.ch/protter/), a web application to visualize the sequence, annotations, and topology of the individual proteins, has been applied to visualize the amino acid residues of ACE2 and domains [16]. The signaling network of ACE is curated by the SIGNOR (SIGnaling Network Open Resource) v.2 [17]. Functional enrichment analysis and gene ontology were retrieved from (https://toppgene.cchmc.org/enrichment.jsp), and gene–gene interaction was retrieved from GeneMania (https://genemania.org/).

## COVID-19 Cell entry

About one-third of the viral genetic content is directed to encode four structural proteins, including spike glycoprotein (S), a small envelope protein (E), matrix protein (M), and the nucleocapsid protein (N) [18]. The glycoprotein (S) of the virus consists of two subunits named S1 and S2 [19]. S1 is mainly responsible for the virus-host interaction and cellular tropism with the critical function domain-receptor-binding domain (RBD), and S2 facilitates virus cell/host cell membrane fusion [20]. The infectivity assays on HeLa cells with or without ACE2 proteins extracted from bats, civets, pigs, mice, and humans, revealed that SARS-CoV-2 uses ACE2 to promote its entry into ACE2-expressing cells, but not from mouse species. It cannot enter those cells without ACE2, which can be considered specific receptors for this virus resembling SARS-CoV [21]**.** Additionally, the previous investigators excluded other receptors suspected of SARS-CoV-2 cell invasion, like dipeptidyl peptidase 4 and aminopeptidase N.

**Fig. 1:**
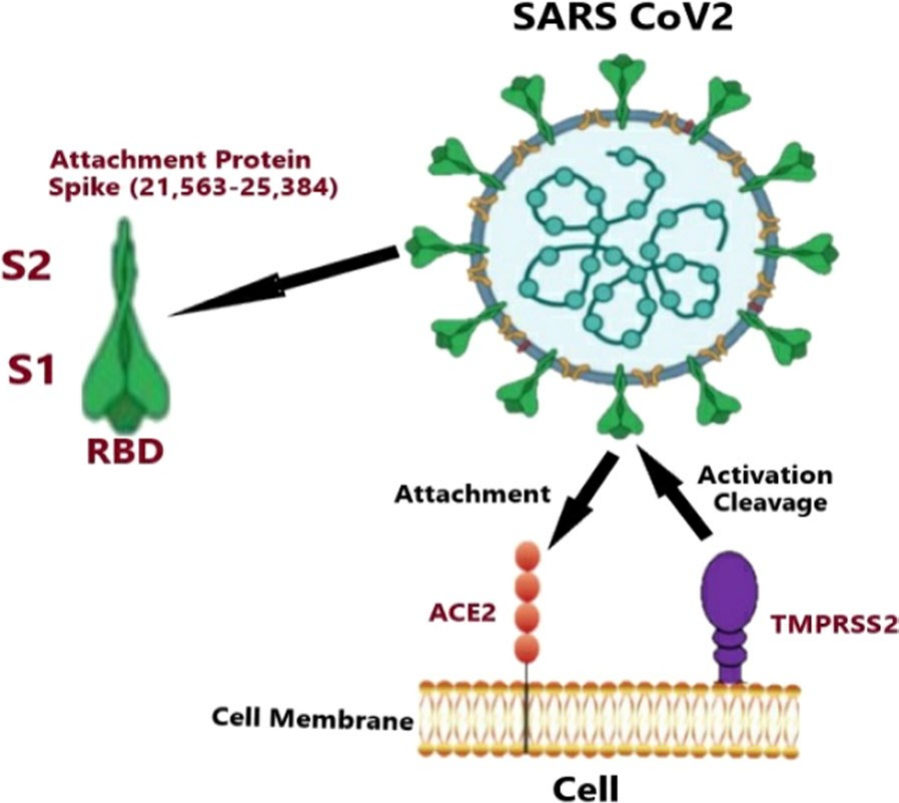
SARS-CoV-2 cellular entry. The binding between the S-protein trimer of SARS-CoV-2 and ACE2 receptor. The figure demonstrated the receptor-binding domain (RBD) of the S1 subunit and the hydrophobic fusion peptide of the S2 subunit where the S-protein is bound to ACE2 and primed with the cellular protease TMPRSS2, and the fusion S2 subunit undergoes a conformational rearrangement. The affinity between ACE2 and the RBD of SARS-CoV-2 is higher than its affinity with the RBD of the SARS virus [27–29]

Coronavirus spike (S) glycoproteins facilitate viral entry and replication into cells through binding to ACE2 and its priming by the serine protease TMPRSS2 (Fig. 1) [22]. Tai and his research team discovered the presence of RBD in the SARS-CoV-2 S1 subunit and observed a robust binding ability to ACE2; moreover, they showed a significantly higher binding affinity than that to SARS-CoV, which may explain the higher infectious rate of SARS-CoV-2 over SARS-CoV [23, 24]. It was also found that the temperature-sensitivity for the SARS-CoV-2 binding affinity is much more than that for SARS-CoV, predicting that the SARS-CoV-2 infection rate would reduce with increased temperature much quicker than SARS-CoV [23]. Also, S glycoproteins give sanctuary to a furin cleavage site which enhances cell invasion and is considered a unique feature for SARS-CoV2 and could be targeted for antibodies [25]. S ectodomain trimer could be a beneficial target for designing vaccines and antiviral entry inhibitors. It was documented that murine polyclonal antibodies against SARS-CoV S effectively diminished SARS-CoV2 S mediated cell entry; this emphasizes the cross-neutralizing antibodies' role in conserving S epitopes upon vaccination [25]**.**

Although SARS-CoV-2 does not group inside SARS and SARS-related coronaviruses, structural investigation distinguished residues in the SARS-CoV-2 RBD that are basic for ACE2 binding; most of them share analogous side chain with that in the SARS-CoV RBD. Such structural similarity and succession unequivocally contend for the evolution between the SARS-CoV-2 and SARS-CoV RBDs to improve the binding ability to ACE2 receptors [26].

## Angiotensin-converting enzyme 2 (ACE2): the hottest target of SARS-CoV-2 invasion

**Fig. 2:**
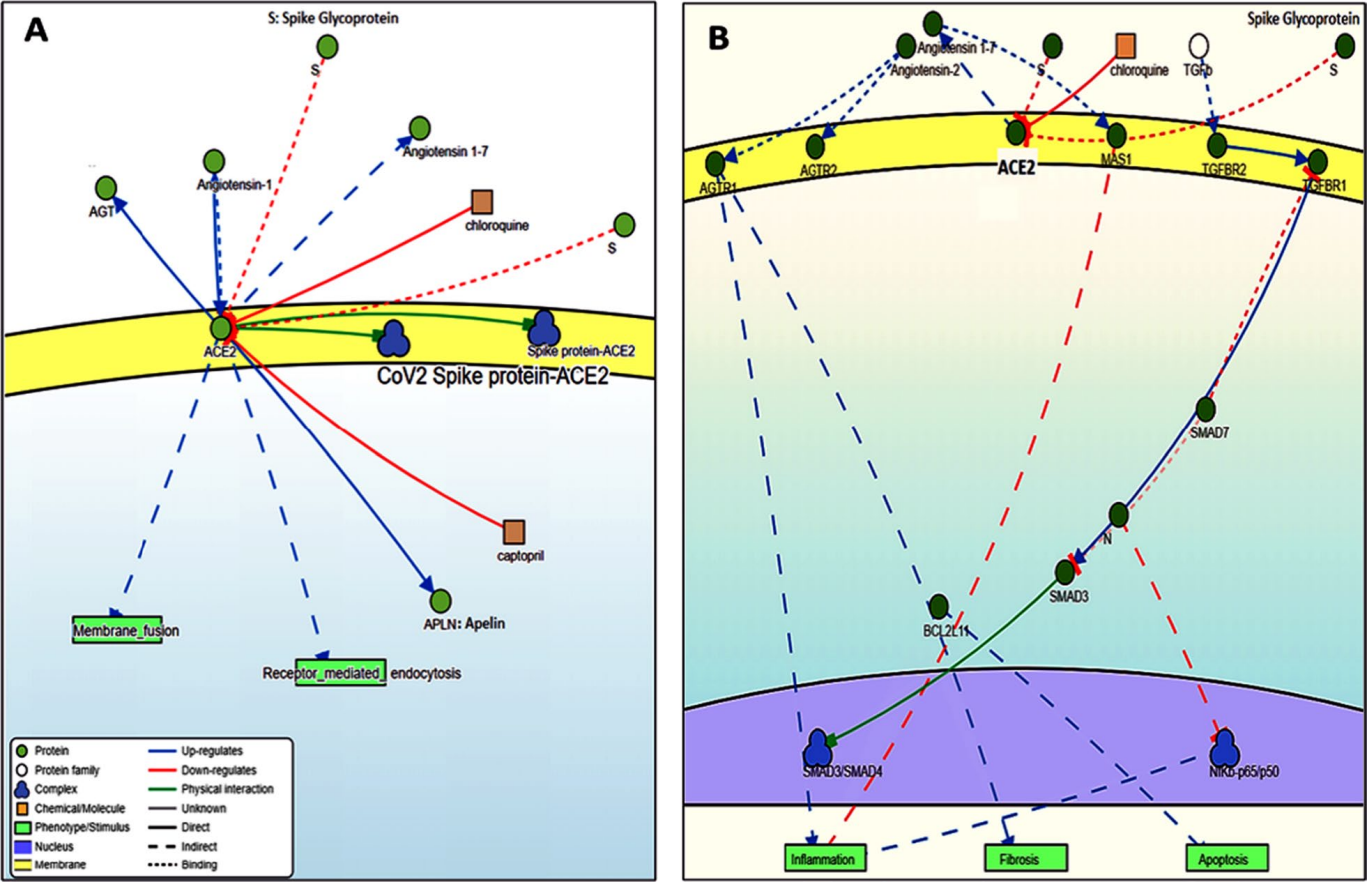
ACE2 signaling network. **A** ACE2 interacts with several molecules, including S-protein. **B** The role of ACE2 in lung fibrosis. TGF-β1 is an essential mediator of fibrosis by activating downstream “SMAD” signaling, which triggers pro-fibrotic genes overexpression. Smad3 is the central canonical mediator for fibrogenesis, while Smad7 negatively regulates the fibrosis process. The RAS also controls the fibrotic process. Down-regulation of ACE2 decreases the angiotensin 1–7 level, upregulating the AT1R signaling cascade, which in turn triggers fibrosis (Data source: https://signor.uniroma2.it/)

ACE2 (EC:3.4.17.23) is also termed as angiotensin-converting enzyme homolog (ACAH), ACE-related carboxypeptidase, and metalloprotease 15 (MPROT15), and was identified as the first reported ACE homolog in 2000. The protein is related to the ACE family of dipeptidyl-carboxypeptidases, which converts angiotensin I to angiotensin 1–9, and angiotensin II to angiotensin 1–7, which acts as a vasodilator and exerts important modulatory effects on the cardiovascular system [30–32] also effectively hydrolyzes apelin-13 and dynorphin-13 [32] (Fig. 2A). By cleavage, angiotensin II may be an essential regulator of heart function and may also have a protective role in acute lung injury [30, 31] (Fig. 2B). Furthermore, it plays a vital role in amino acid transport by acting as a binding partner of amino acid transporter SL6A19 in the intestine, regulating trafficking and the expression on the cell surface, and its catalytic activity [33].

**Fig. 3:**
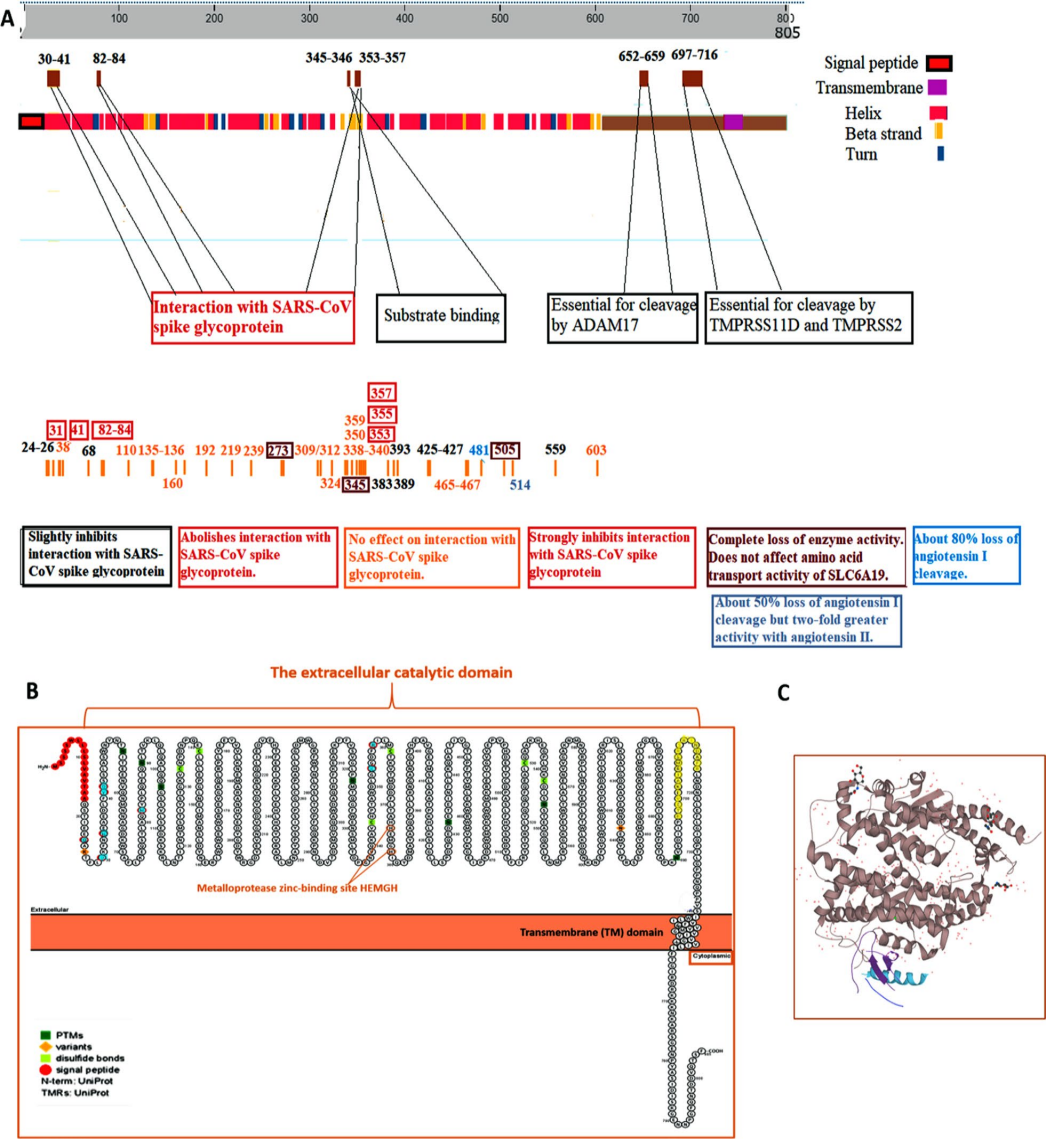
Sequence and aa residues of ACE2. **A** *ACE2* gene encoding a deduced 805-aa protein. The unprocessed ACE2 (1–17) and ACE2 chain (18–805). Functional roles are illustrated based on [34] and [35]. (https://www.uniprot.org/uniprot/Q9BYF1/protvista). **B** ACE2 sequence contains an N-terminal signal (1 to 18), a TM domain (740 to 763), and a metalloprotease zinc-binding consensus (374 to 378, HEMGH). Blue aa residues are required for RBD interaction with the extracellular catalytic domain of ACE2 [36]. **C.** 3D structure of the ACE2 protein (https://www.uniprot.org/uniprot/Q9BYF1/protvista)

ACE2 is a metalloproteinase with a total length of 805 amino acids (Fig. 3A) [34, 35]. It belongs to type I transmembrane glycoprotein (integral) and contains a single protruding extracellular catalytic domain. Like ACE, ACE2 has two domains: an amino-terminal catalytic domain and another carboxy-terminal domain. The catalytic domain has an active site called the zinc metallopeptidase domain (HEXXH motif) (Fig. 3B) [36].

Based on a recent study by Yan et al., the RBD Gln 498, Thr 500, and Asn 501 of the SARS-CoV-2 configure a connecting net of hydrogen bonds with ACE2 structured Tyr 41, Gln 42, Lys 3535, and Arg 357, respectively. Furthermore, Lys 417, Tyr 453, and Gln 474 of RBD interact with Asp 30, His 34, and Gln 24 of ACE2, respectively. Through Vander Waals forces, Phe 486 of RBD interacts with Met 82 of ACE2 to ensure binding of the virus to the receptor and subsequent internalization (Fig. 3) [36]. Interestingly, the TMPRSS2 cleaves the ACE2 residues 697 to 716 to facilitate the S-protein-driven viral entry [37]. The impact of some experimental mutation of one or more amino acids on the biological properties of the ACE2 protein, in particular, the binding to SARS-CoV (by similarity could be SARS-CoV2), is summarized in Table 1 [33, 35, 38, 39]. It is worth noting that ACE2 can also be trimmed by “a disintegrin and metalloproteinase domain-containing protein 17 (ADAM 17),” which releases an extracellular fragment called soluble ACE2 (sACE2) and is measured as sACE2 plasma activity [40]. It has been considered a possible candidate for monitoring the evolution of COVID-19 [41]. The sACE2 retains an intact SARS-CoV-2 interaction site, suggesting its ability to bind to SARS-CoV-2. Kornilov and colleagues have observed that COVID-19-related regulatory pathways may induce ACE2 shedding, and the sACE2 concentrations may correlate with the level of systemic inflammation associated with COVID-19 [42]. Furthermore, the calmodulin–calcium signaling pathway which contributes to ACE2 release has been suggested to add new insights for clinical/therapeutic applications of ACE2 for COVID-19 [43].

**Table 1:** Effect of the experimental mutation of one or more amino acid(s) of ACE2 on the biological properties of the protein

Amino acid(s) position	Description	
24 – 26	QAK → KAE: Slightly inhibits interaction with SARS-CoV spike glycoprotein	1
31	K → D: Abolishes interaction with SARS-CoV spike glycoprotein	1
37	E → A: No effect on interaction with SARS-CoV spike glycoprotein	1
38	D → A: No effect on interaction with SARS-CoV spike glycoprotein	1
41	Y → A: Strongly inhibits interaction with SARS-CoV spike glycoprotein	1
68	K → D: Slightly inhibits interaction with SARS-CoV spike glycoprotein	1
82 – 84	MYP → NFS: Inhibits interaction with SARS-CoV spike glycoprotein	1
110	E → P: No effect on interaction with SARS-CoV spike glycoprotein	1
135 – 136	PD → SM: No effect on interaction with SARS-CoV spike glycoprotein	1
160	E → R: No effect on interaction with SARS-CoV spike glycoprotein	1
169	R → Q: About 95% loss of angiotensin I cleavage	2
192	R → D: No effect on interaction with SARS-CoV spike glycoprotein	1
219	R → D: No effect on interaction with SARS-CoV spike glycoprotein	1
239	H → Q: No effect on interaction with SARS-CoV spike glycoprotein	1
271	W → Q: About 95% loss of angiotensin I cleavage	2
273	R → Q: Complete loss of enzyme activity. Does not affect the amino acid transport activity of SLC6A19	3, 4
309	K → D: No effect on interaction with SARS-CoV spike glycoprotein	1
312	E → A: No effect on interaction with SARS-CoV spike glycoprotein	1
324	T → A: No effect on interaction with SARS-CoV spike glycoprotein	1
338 – 340	NVQ → DDR: No effect on interaction with SARS-CoV spike glycoprotein	1
345	H → A: Complete loss of enzyme activity	3
350	D → A: No effect on interaction with SARS-CoV spike glycoprotein	1
353	K → H, A or D: Abolishes interaction with SARS-CoV spike glycoprotein	1
355	D → A: Strongly inhibits interaction with SARS-CoV spike glycoprotein	1
357	R → A: Strongly inhibits interaction with SARS-CoV spike glycoprotein	1
359	L → K or A: No effect on interaction with SARS-CoV spike glycoprotein	1
383	M → A: Slightly inhibits interaction with SARS-CoV spike glycoprotein	1
389	P → A: Slightly inhibits interaction with SARS-CoV spike glycoprotein	1
393	R → A: Slightly inhibits interaction with SARS-CoV spike glycoprotein	1
425 – 427	SPD → PSN: Slightly inhibits interaction with SARS-CoV spike glycoprotein	1
465 – 467	KGE → QDK: No effect on interaction with SARS-CoV spike glycoprotein	1
481	K → Q: About 80% loss of angiotensin I cleavage	2
505	H → A: Complete loss of enzyme activity	3
514	R → Q: About 50% loss of angiotensin I cleavage but twofold greater activity with angiotensin II	2
559	R → S: Slightly inhibits interaction with SARS-CoV spike glycoprotein	1
603	F → T: No effect on interaction with SARS-CoV spike glycoprotein	1

Data source: https://www.uniprot.org/uniprot/Q9BYF1

## Structural and functional analysis of *ACE2*

**Fig. 4:**
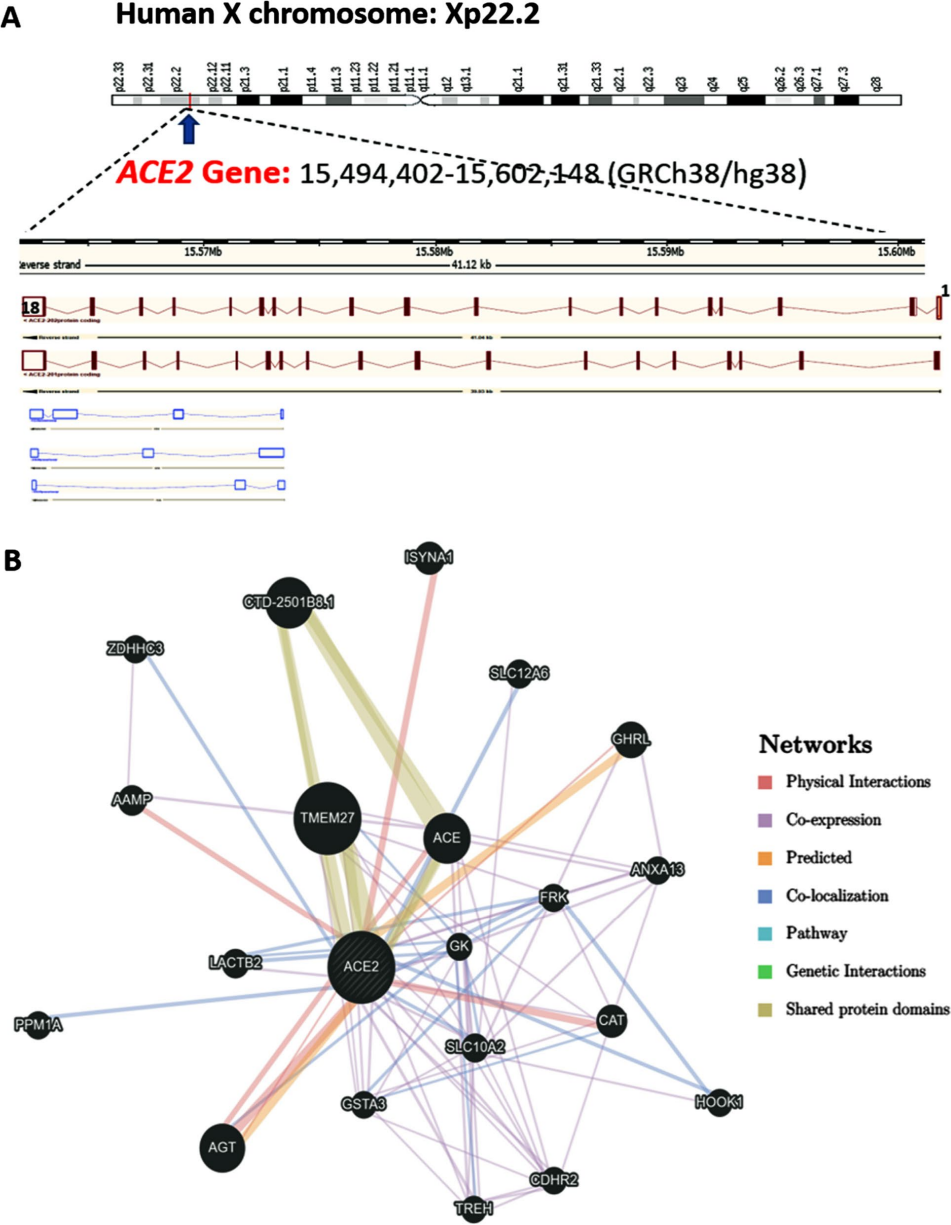
*ACE2* genomic structure and interactions. **A** The *ACE2* gene is mapped to X chromosome Xp22.2. It contains 18 exons and has five transcripts; from the top to down: protein-coding transcripts ACE2-202 (3507 bp; 805aa) and ACE-201 (3339 bp; 805aa), in addition to three noncoding processed transcripts; ACE2-203/4/5 (998/786/599 bp, respectively). **B** Gene–gene network analysis for *ACE2* gene. (https://genemania.org/search/homo-sapiens/ACE2/)

Human *ACE2* (NCBI_Gene ID:59,272), a protein-coding gene, is located along the short arm of the Chromosome X (Xp22.2), spanning 41,116 bases long on the reverse strand from 15,561,033 to 15,602,148, according to the “Human Genome Assembly GRCh38” (Fig. 4A). It comprises 18 exons that can be transcribed into five different splice variants (ACE2-201 to ACE2-205); only two are protein-coding, as depicted in Fig. 4A [44].

A recent study by Fujikura and Uesaka REF has identified 349 single nucleotide variants (SNVs) in the coding regions and splice sites. SNVs were found in multiple protein-coding regions, including those in the contact residues between SARS-COV2 and human ACE2. There were 247 missense SNVs (70.8%) and 94 synonymous SNVs (26.9%). The residual 2% of SNVs, stop-gained (*n* = 2), splice site variants (*n* = 2), start-loss (*n* = 1), and indels (*n* = 3) were recorded. The majority of these SNVs were rare or quite rare, with allele frequency < 1% or < 0.001%, respectively. The frequency of deleterious SNVs is higher for rare SNVs than for SNVs with a high allele frequency [45].

Gene–gene network analysis reveals the implication of ACE2 in angiotensin maturation, regulation of systemic arterial blood pressure, peptide hormone metabolism, proteolysis, and regulation of cytokine production, among others (Fig. 4B).

**Fig. 5:**
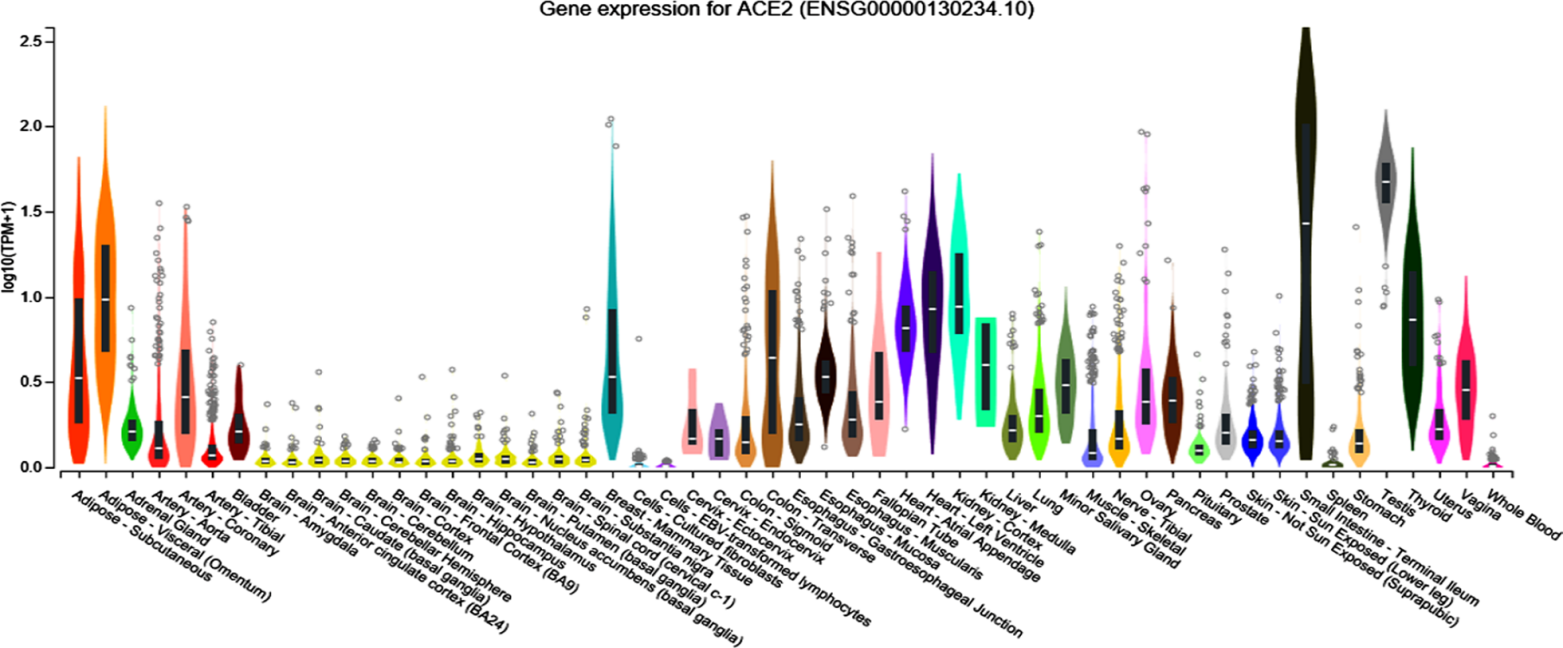
ACE2 expression in different human tissues. Expression values are shown in transcripts per Million (TPM), calculated from a gene model with isoforms collapsed to a single gene. Each box plot represents the median, 25th percentile, and 75th percentile. The points below or above 1.5 times the interquartile range are considered outliers. Data Source: GTEx Analysis Release V8 (dbGaP Accession phs000424.v8.p2) [51]

There is an expression of this gene in endothelial cells in small/large arteries, arterial smooth muscle cells, the heart, the alveolar epithelial cells, the small intestine enterocytes, Leydig cells, and Sertoli cells [46–50] (Fig. 5). Recently, it was discovered to be expressed in the proximal renal tubules and the small intestine [51]. According to its organ- and cell-specific expression, this gene regulates both cardiovascular and renal function, in addition to fertility (https://www.ncbi.nlm.nih.gov/gene/59272).

**Fig. 6:**
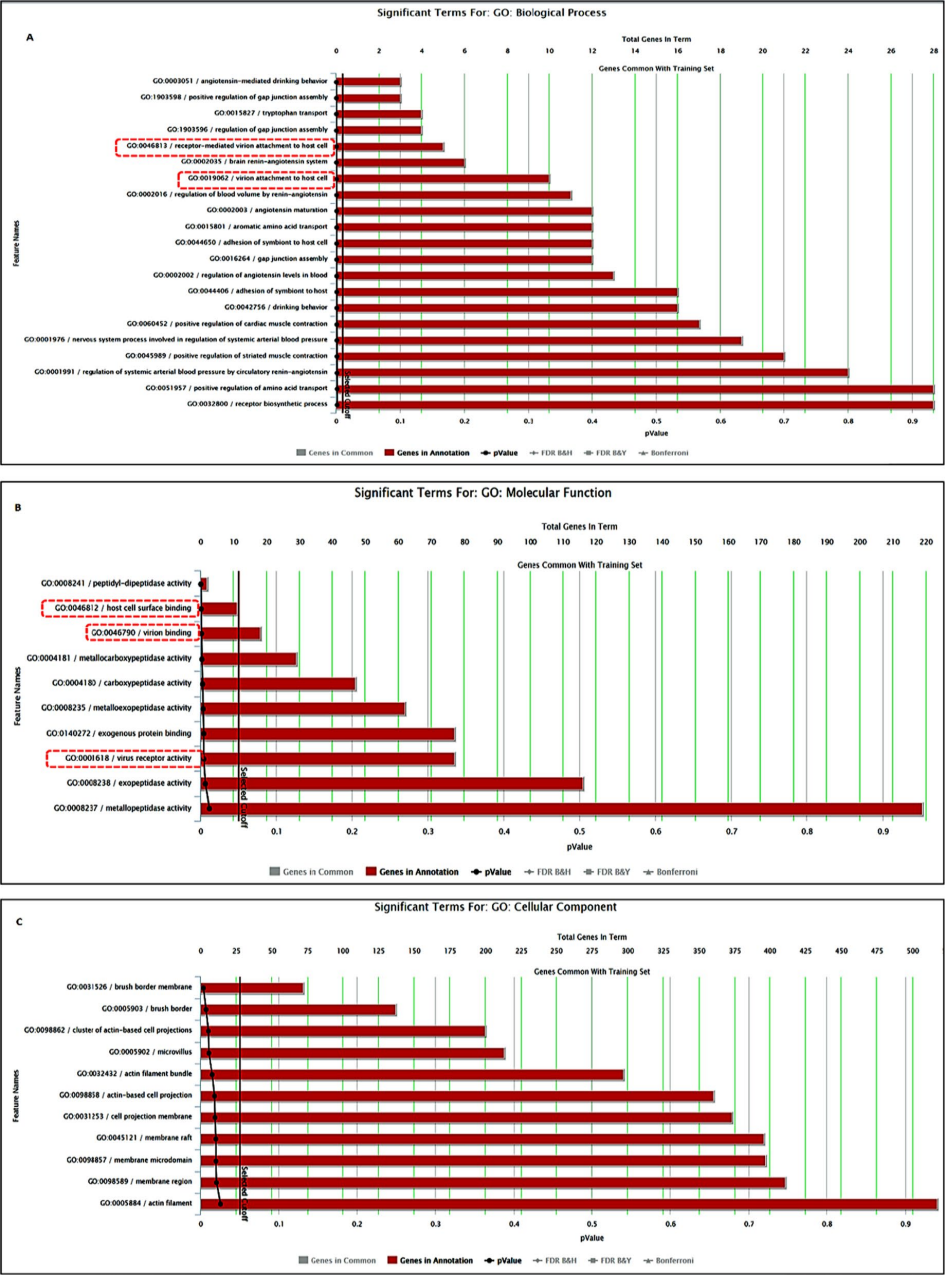
Functional annotation of *ACE2*. **A** Biological process, **B** Molecular functions, and **C** Cellular components for ACE2 with *p*-values set at < 0.01 for (A) and < 0.05 for (B and C). The processes and molecular functions related to coronavirus binding and infection are encircled by red broken line rectangles (https://toppgene.cchmc.org/)

Gene ontology (GO) annotations related to this gene include virion attachment to host cell (receptor-mediated) among others in biological process category (Fig. 6A), host cell surface binding, virion binding, and virus receptor activity in the molecular function category (Fig. 6B), membrane region and raft, cell projection membrane, microvillus, and brush border membrane in the cellular component group (Fig. 6C).

## Association of *ACE2* gene variants with SARS-CoV2 infection

Since virus receptors are essential for cellular pathogen entry, they can influence the development and/or progression of viral diseases [52]**;** previous studies tried to determine whether the *ACE2* variants and/or expression could be associated with SARS-CoV/SARS-CoV2 with conflicting results.

Although an earlier report demonstrated no association between *ACE2* variants and SARS-CoV susceptibility or outcomes with no difference related to sex [13], Gemmati et al. reported a higher incidence of COVID-19 infection in males with more severe representations. Death rates from SARS-CoV2 infection were 65 percent higher in males than in females. Part of the reason for these observations is the location of ACE2 on chromosome Xp22.22. This X-linked association renders the heterozygous females with higher ACE2 expression more protected than the hemizygous males [53].

Similarly, through analysis of the “1000 Genomes Project,” which contains samples from almost all ethnicities, a study has suggested the possibility of “population-specific” *ACE2* genetic variations that impact the susceptibility to SARS-CoV2 infection [54]. Also, the genetic analysis by Cao and co-workers did not find any mutation difference in *ACE2* that would influence SARS-CoV2/ S-protein binding [15]. Cao et al., however, were criticized by some researchers for focusing merely on a limited population variation data set [52]. Using data mining in several data sets and applying “structural predictions,” Suryamohan et al. could predict several *ACE2* variants (i.e., “*E23K*, *S19P*, *I21V*, *K26R*, *T27A*, *N64K*, *T92I*, *K26E*, *H378R*, *Q102P*, and *M383T*”) which have the potential to increase the sensitivity of the host to SARS-CoV. Alternatively, “*N33I*, *K31R*, *D38V*, *H34R*, *E35K*, *E37K*, *N51S*, *K68E*, *Y50F*, *F72V*, *G326E*, *G352V*, *Y83H*, *D355N* and *Q388L*” variants were predicted to decrease S-protein-ACE2 binding affinity with a subsequent decline in infection susceptibility [55]. Interestingly, most of the previously predicted variants were clustered in the N-terminal region (extracellular catalytic domain) of ACE2 (Fig. 3B) that interacts with the S-protein. However, the latter investigators confirmed that the above-identified variants are present in the general population with rare allele frequencies without any significant observable frequencies among different populations or even when stratified by sex. Another Italian research group has explored some *ACE2* variants that could impact protein stability and SARS-CoV-2 binding. They found that c.1517 T > C p. (Val506Ala) had the highest disturbance effect, c.631G > A p.(Gly211Arg) and c.77A > G p. (Lys26Arg) had a high frequency of allele as well as c.1166C > A p.(Pro389His), and c.1051C > G p.(Leu351- Val) were predicted to affect the interaction of spike protein [14]. Furthermore, through comparative genetic analysis of nearly 81,000 human genomes across eight populations, Hou et al. explored 63 potentially deleterious *ACE2* variants that could affect the genetic susceptibility to COVID-19 [56].

## *ACE2* gene variants and COVID-19 outcome

The *ACE2* gene variants may modify the deleterious consequences of SARS-CoV2 to the host cells [56, 57]. The first COVID-19 genome-wide association study identified the 3p21.31 gene cluster, including “*SLC6A20*, *LZTFL1*, *CCR9*, *FYCO1*, *CXCR6*, and *XCR1*” as a genetic susceptibility locus in severe patients with COVID-19 and respiratory failure [58]. Previous genetic studies indicated that *ACE2* polymorphisms are related to the rate of hypertension progression in different populations [59]. *ACE 2* variants were also found to be associated with cardiovascular and pulmonary conditions through altering the angiotensinogen-ACE2 interactions [56]. Several mutations have been speculated to modify the ACE2 protein expression level, as reported previously in a murine model [60]. Also, *ACE2* deletion in the mice model was associated with increased tissue/circulation Ang II levels and cardiovascular damage [61, 62]. The mechanisms by which *ACE2* gene variants could impact the structural and/or the catalytic activity of the gene product could be at the transcriptional (mRNA expression), post-transcriptional modifications (such as N-glycosylation), or ACE2 protein levels that influence the outcome of COVID-19 by acting on blood pressure through the RAS and possible impact on lung/heart damages through the Ang II-triggered oxidative stress [44].

Furthermore, the recent study by Khayat et al. has unraveled at least ten *ACE2*-related variants in coding, noncoding, and regulatory sites that can offer a plausible biological explanation for the epidemiological differences related to COVID-19 [57]. They have identified the rs182366225 and rs2097723 variants associated with ACE2 upregulation to be more prevalent (30% to 180% more frequently) in the East Asian population, whereas rs1027571965 and rs889263894 variants were exclusively found in indigenous populations from Amazon. In contrast, the later population had higher frequencies of “rs2285666 and rs35803318” than other populations. Furthermore, Africans were identified to have higher rates of three relevant polymorphisms (rs147311723, rs142017934, and rs4646140), in which “rs142017934” was exclusive to this population and associated with gene upregulation. However, Europeans and some Africans have a higher frequency of an (rs5934250) allele that seems to downregulate *ACE2* in some tissues [57]. The ACE insertion/deletion (I/D) variant, which influences enzyme levels with subsequent change in the “ACE/Ang II/AT1R axis” function, also showed many correlations with SARS-CoV-2 infection and appeared to impact the outcome of COVID-19 disease [4]. It has been observed that the (II) genotype (that is associated with the least ACE plasma levels compared to the ID/DD variants) is the most prevalent genotype among asymptomatic COVID-19 cases. However, the (DD) genotype is predominate in COVID-19 patients, particularly in the European elderly population who present with severe disease phenotype and could increase the risk of COVID-19-related mortality [63, 64]. More detailed associations of ACE I/D variant with COVID-19 severity and comorbidities have been covered in the interesting review by Gintoni et al. [4].

## *ACE2* gene expression and COVID-19 outcome

Higher *ACE2* expression was reported in men's lungs more than women, while serum activities appear higher in females than males, supporting the hypothesis related to the observed gender-related differences in disease severity/outcome [65–67]. The putative role of estrogen in upregulating the *ACE2* expression/plasma activity was suggested as a possible cause for relative female protection against COVID-19 infection compared to males [68]. Also, given the site of *ACE2* locus on the X chromosome, these could explain in part the severe phenotype of COVID-19 in males compared to females [69]. Moreover, several differences in the ACE2 expressions have been observed between different countries, which correlate with genetic variations[70, 71]. *ACE2* expression in Asian individuals was reported to be more significant, in healthy human lung samples, than in Caucasians and African Americans [12, 72].

Similarly, an *ACE2* quantitative expression analysis study on East Asians, Europeans, Africans, South Asians, and mixed Americans reported higher expression levels of *ACE2* in East Asians [15] that could partly explain the variations in disease outcome among different populations. Osman et al. reported a decrease in the expression of circulating *ACE2* mRNA and cell surface ACE2 during COVID-19, and prolonged viral shedders of COVID-19 were associated with low sACE2 plasma concentrations [41]. As a result, they concluded that ACE2 no longer metabolizes Ang II with increased plasma concentrations associated with worse outcomes. Furthermore, the soluble forms of ACE2 have recently been shown to inhibit SARS-CoV-2 infection [43]. In the context of enhanced ACE2 deficiency produced by the viral invasion, the significant “ACE2/AT1-7/Mas axis” dysregulation could contribute to augmenting the inflammatory/thrombotic processes progression [73]. Even the ACE2 expression/activity has been found to change rapidly in response to certain food items [74] and many food components are reported to be useful for the treatment of COVID-19, and these may act through altering ACE2 expression and/or its activity as detailed in the recent Sahu et al. review article [67].

Wooster et al. have suggested in their preprint article that five *ACE2*-related variants “rs4240157, rs6632680, rs4830965, rs1476524, and rs2048683” might be associated with higher *ACE2* tissue-specific expression, resulting in hospitalization, whereas the “rs1548474” variant showed association with low tissue expression and lesser severity [75]. Also, the rs2106809 variant has been suggested to be associated with variable circulating ACE2 levels, whereas CC/CT genotype resulted in greater levels when compared with the TT genotype. Therefore, “quantification of soluble ACE2 (sACE2) in body fluids was suggested as a protective biomarker for a rapid test screening,” as concluded by Chaudhary [40]. Additionally, a combined effect of genetic variants in genes responsible for the synthesis of proinflammatory cytokines/chemokines along with *ACE2* has been suggested to be responsible for differences in patients' response to COVID-19 in terms of hypercytokinemia/cytokine storm that characterized by excessive proinflammatory cytokine production associated with multiple organ failure [76].

## ACE2 role in emerging COVID-19-related treatment

In seeking a suitable treatment for COVID-19, recently, the RBD of SARS-CoV-2 spike glycoprotein (S-protein) was modeled in 242 structural models with variations of human ACE2 binding [77]. Several *ACE2* variants have been speculated in the African and American populations, including “p.Met383Thr, p.Pro389His, and p.Asp427Tyr,” which may influence the clinical efficacy of hydroxychloroquine or chloroquine [56]. This could explain why therapeutic use of hydroxychloroquine was not significantly associated with differences in “in-hospital mortality” [78].

Also, one of the proposed strategies for COVID-19 treatment was the soluble ACE2 and ACE2-Fc fusion protein that work as decoy receptors to SARS-CoV2 [79]. Using “Clinical-Grade sACE2,” in vitro study showed that the human recombinant soluble ACE2 (hrsACE2) could significantly block early stages of SARS-CoV-2 infections [80]. Also, it has been suggested that designing a recombinant non-functioning form of sACE2, which carries one or more of the specified variants that show a gain of function activity and permit binding to the viral RBD more avidly, could have a potential virus neutralization and COVID-19 treatment [79]. Similarly, by using functional models and molecular dynamics simulations, Zhang et al. could point to the broad efficacy of an engineered sACE2 decoy (has three amino acid substitutions) against SARS-CoV-2 variants in mice by markedly augmenting the affinity for the S-protein of several SARS-CoV-2 variants, supporting its therapeutic potential [81]. Recently, Vitiello and Ferrara demonstrated the significant pharmacological synergism of the triple therapy baricitinib (immunomodulator)/remdesivir (antiviral)/rhACE2 (a soluble recombinant human form of ACE2) for the effective treatment of COVID-19. The “rhACE2” could activate the Ang 1–7 and Ang 1–9 biosynthesis pathway of the RAS system by decreasing Ang II levels; this could be associated with a decline in cytokine proinflammatory concentration [82]. Thus, the rhACE2 could prove “useful as a trap effect for circulating SARS-CoV2 and decrease viral load and hinder infection,” as the investigators concluded [82].

Interestingly, El-Shennawy et al. reported “an increase about 135-fold higher potency in blocking the binding of the viral spike protein RBD, and a 60- to 80-fold higher efficacy in preventing infections by SARS-CoV-2” for their newly identified circulating extracellular vesicles that express ACE2 (evACE2) compared to vesicle-free rhACE2 [83]. They proved that evACE2 could protect the hACE2 transgenic mice from SARS-CoV-2-induced lung injury and mortality and proposed its application as a treatment modality to existing and/or future coronaviruses that use ACE2 receptors. Another therapeutic modality based on the potential use of the intranasal “ACE2-overexpressing A549 cell-derived microparticles (AO-MPs)” that are taken up by alveolar macrophages, in which these particles increase the endosomal pH with a decrease in the lysosomal pH in these cells, thus directing the bound SARS-CoV-2 from phago-endosomes to lysosomes for subsequent degradation. In this way, these particles could also inhibit the proinflammatory phenotype of the alveolar macrophages, increasing the treatment efficacy against the virus in the mice model with few (if any) side effects [84].

Another emerging proposal for COVID-19 treatment has been assumed by Bakry et al., in which they suggested the use of the mesenchymal stem cells that are coated with anti-ACE2 antibodies to help in the achievement of better cell attachment to SARS-CoV2- infected cells and competing with the virus for the same receptor [85]. They proposed that the attached antibodies be targeted to the metallopeptidase domain (19–611 a.a.) of ACE2 that interacts with the S-protein. Additionally, Wang et al. developed an “inhaled microfluidic microsphere” with a genetically engineered membrane from ACE2 receptor-overexpressing cells/macrophages. As this system competes with the virus for ACE2 binding, it can significantly reduce the viral infectivity along with the respiratory system *in vitro* and *in vivo,* as well as can efficiently alleviate the proinflammatory cytokine storm [86]. Although all the studies mentioned above open a new era in COVID-19 treatment and management, Hou et al. recommended that “further pharmacogenomic studies that integrate drug response and genetic data from patients with COVID-19 are urgently needed” [56] to help future targeted and personalized therapy applications in clinics.

## Conclusions

It is worth noting that apart from the differences in *ACE2* genetic variations and gene expression, many other genetic and/or environmental factors, including, for example, the genes related to the innate and adaptive immunity, the viral load, the preventive precautions that are taken at the level of the individuals and the countries, among others, could influence COVID-19 virulence and modify disease outcome. Most of the studies mentioned above have limitations, including the non-reproducibility of genetic variant studies among different ethnic groups [40]. So much is yet to be known.

## Data Availability

The authors declare that all data used to elaborate this project is available for consultation by emailing the correspondent author.

## Abbreviations

ACE2: Angiotensin-converting enzyme 2
AAs: Amino acids
ACAH: Angiotensin-converting enzyme homolog
AECII: Alveolar epithelial type II cells
AO-MPs: ACE2-overexpressing A549 cell-derived microparticles
E: A small envelope protein
evACE2: Extracellular vesicles that express ACE2
hrsACE2: Human recombinant soluble ACE2
I/D: Insertion/deletion
M: Matrix protein
MPROT15: Metalloprotease 15
N: Nucleocapsid protein
RAS: Renin-angiotensin system
RBD: Receptor-binding domain
S: Spike glycoprotein
sACE2: Soluble ACE2
SARS-COV: Severe acute respiratory syndrome coronavirus
SARS-COV-2: Severe acute respiratory syndrome coronavirus 2
SIGNOR: SIGnaling network open resource
Smad: Small mother against decapentaplegic
TGF-β1: Transforming growth factor-β1
TMPRSS: Transmembrane protease, serine 2
